# Colonization with Multidrug-Resistant Bacteria in the First Week of Life among Hospitalized Preterm Neonates in Serbia: Risk Factors and Outcomes

**DOI:** 10.3390/microorganisms9122613

**Published:** 2021-12-17

**Authors:** Marija Milic, Marina Siljic, Valentina Cirkovic, Milos Jovicevic, Vladimir Perovic, Milos Markovic, Jelena Martic, Maja Stanojevic, Vera Mijac

**Affiliations:** 1 Department of Neonatal Intensive Care, Institute of Neonatology, Kralja Milutina 50, 11000 Belgrade, Serbia; marija.milic78@gmail.com; 2 Department of Bacteriology, Virology and Immunology, Institute of Microbiology and Immunology, Faculty of Medicine, University of Belgrade, Dr Subotica 1, 11000 Belgrade, Serbia; marinasiljic@gmail.com (M.S.); valentinanikolic85@gmail.com (V.C.); jovicevic.milos@gmail.com (M.J.); perovic.vladimir@gmail.com (V.P.); maja.stanojevic@med.bg.ac.rs (M.S.); 3 Department of Neonatology, Institute for Mother and Child Health Care of Serbia “Dr Vukan Cupic”, Faculty of Medicine, University of Belgrade, Radoja Dakica 6, 11070 Belgrade, Serbia; jelena@net2yu.net

**Keywords:** preterm neonates, multidrug-resistant bacteria, gut colonization, risk factors, infection, outcome

## Abstract

The aim of this prospective cohort study was to determine the prevalence of gut colonization with multidrug-resistant (MDR) bacteria, risk factors for colonization, infection risk, and outcomes among preterm neonates hospitalized at a tertiary-care center in Serbia. During the period from December 2017 to April 2018, 103 neonates were screened for rectal carriage at admission and on the seventh day of life. Characterization of MDR strains was done by conventional microbiology and molecular methods. Out of 61 (59.2%) colonized neonates, 12 (11.6%) were found colonized at admission, while 49 (47.6%) became colonized at the study site. Among a total of 72 MDR isolates, extended-spectrum beta-lactamase (ESBL)-producing enterobacteria prevailed (56/72, 77%), followed by *Acinetobacter baumannii* (14/72, 19%). The majority of ESBL-producing strains carried multiple genes (*bla*TEM/*bla*CTX-M-15 or *bla*TEM/*bla*SHV). Longer previous hospitalization and delivery by cesarean section were associated with MDR colonization, while mechanical ventilation was a risk factor for colonization at the study site. Infections due to MDR bacteria were more frequent among colonized than non-colonized neonates, but not significantly, and mortality was low (1%) in the studied neonates. These results indicate that hospitalized preterm neonates in Serbia are rapidly colonized with a diversity of MDR species and resistance phenotypes/genotypes.

## 1. Introduction

The increasing health threat posed by multi-drug resistant (MDR) bacteria emerged alongside the rise and global dissemination of antimicrobial resistance. Premature neonates (born before 37 weeks of gestation) comprise a vulnerable population often requiring long-term hospitalization due to the overall immaturity, underdeveloped organ systems and need for invasive manipulations. Prematurity is a leading cause of death in children <5 years of age [1], and in many cases due to health-care associated (HAI) infection that develops in the first weeks of life [2,3].

Microbial colonization in preterm neonates often commences in the hospital environment which harbors potentially pathogenic microorganisms, including multidrug-resistant ones. The overall immaturity, delivery mode and other predisposing factors have been shown to increase the risk of MDR acquisition in preterm neonates [4].

Colonization with MDR bacteria may remain unnoticed; nevertheless, it may also predispose the development of infection. In preterm neonates, mucosal barriers are underdeveloped and vulnerable, and thus might allow microbial overgrowth, eventually leading to infection [5]. Furthermore, the connection between gut colonization of neonates and development of subsequent infection has been shown [6] with the risk of infection being highest during the neonatal period (first month of life) [2,7]. MDR Gram-negative bacteria are of special concern due to their increasing prevalence among preterm neonates, the level of antimicrobial resistance, and the lack of therapeutic options [8].

Screening for colonization as an infection prevention practice [9,10] is routinely implemented in health settings with diagnostic capacity and adequate resources, especially in financially-developed countries. Other HAI preventive strategies include hand and environmental hygiene, use of protective equipment, contact precautions, isolation of colonized or infected patients, an adequate nurse-to-patient ratio, antibiotic stewardship, etc. However, many of the abovementioned practices are insufficient in health-care facilities with limited resources.

The spectrum and the prevalence of gut colonizing MDR bacteria among preterm neonates therefore significantly differs between developing countries and high income countries [6,9,11]. Serbia is an upper-middle income country situated in south-eastern Europe, with a recent history of social instability and prolonged economic transition. The available data imply that, in Serbia, bacterial resistance might be widespread in hospital settings [12], but studies of early colonization with MDR bacteria among preterm neonates are lacking.

The aim of this study was to evaluate the prevalence of gut colonization with MDR bacteria among hospitalized preterm neonates in the first week of life, to assess the factors associated with colonization, as well as the risk of developing infection with colonizing microbes during the neonatal period and to investigate the outcomes of infection.

## 2. Materials and Methods

### 2.1. Hospital Settings and Patients

In this prospective cohort study, 103 preterm neonates born in 17 obstetric wards across the country in the period from December 2017 until April 2018 were included. They were randomly selected from a total of 231 neonates admitted to the Institute of Neonatology during this period. Patients’ clinical and demographic characteristics as obtained from medical records are provided in Supplementary Materials Table S1. The parental signed informed consent form was obtained for each participant in the study. The study was carried out at the Institute of Neonatology, which is a tertiary level neonatal center providing long-term health care to premature neonates born in obstetric wards all over the country, with the number of admissions in the range of 700–900 per year. The Institute consists of several units, with the nurse/patient ratio varying from 1:3 to 1:6 in the intensive care unit, and 1:6 to 1:15 in other units based on the number of patients and patients’ condition. For disinfection, alcohol or alcohol-based combinations are used on the skin, for personnel hand hygiene and daily medical devices and surfaces disinfection, while quaternary ammonium compounds in combination with formic acid and/or alcohol are used for floors. Routine protocol on admission included taking blood samples for haemoculture and initiating antibiotic therapy (ampicillin plus amikacin/gentamicin, further referred to as initial therapy) over a duration of 5 days. Additionally, for neonates weighing less than 1600 g and for all patients in the neonatal intensive care unit (NICU), standard admission protocol included the placement of an umbilical vein catheter. Enteral feeding was introduced at the earliest point, with the milk from a donor milk bank, the mother’s milk or preterm formula. Both milk from the donor milk bank as well as mother’s milk are routinely pasteurized and cultured thereafter, and only milk with negative post-pasteurization culture results were given to preterm neonates. In addition, a probiotic strain of *Lactobacillus rahmnosus* GG was administered to all patients during hospitalization. If systemic infection or pneumonia was suspected at any time during the stay of the neonates in the hospital, a blood culture was taken, as well as a tracheal aspirate if appropriate. In the case of two different patients with blood stream infection due to the same pathogen, patient isolation and strict epidemiological measures were introduced (e.g., sampling of surfaces, incubators and healthcare workers hands), aiming to contain further spread of infection. However, screening of colonization at admission and/or during hospitalization was not implemented as a routine practice in the Institute of Neonatology.

### 2.2. Sample Collection

A rectal swab was taken from each patient, placed in Amies transport medium and transported to the laboratory within 12 h. Samples were taken at hospital admission and on the seventh day of life.

### 2.3. Isolation, Identification of MDR Isolates and Antimicrobial Susceptibility Testing

CHROMID ESBL agar (BioMerieux, Marcy l’Etoile, Lion, France) was used for the detection of MDR Gram-negative bacteria, as this medium supports the growth of Gram-negative bacteria with cephalosporin resistance due to extended-spectrum beta-lactamase (ESBL) production, as well as other mechanisms [13]. CHROMID VRE (BioMerieux, Marcy l’Etoile, Lion, France) agar was utilized for the detection of vancomycin-resistant enterococci (VRE), and isolates were identified according to the manufacturers’ instruction. After 24 h and 48 h of incubation in aerobic conditions, media were checked for bacterial growth. Colonies on CHROMID ESBL medium were Gram stained, and for Gram-negative bacilli, identification was done using API 20E (BioMerieux, France) for enterobacteria, and API 20NE for non-fermenting Gram-negative bacilli (BioMerieux, Marcy l’Etoile, Lion, France). Antimicrobial susceptibility testing of all isolates was done with disk diffusion method according to the European committee for antimicrobial susceptibility testing (EUCAST) recommendations [14], with appropriate antibiotic discs (Bio Rad, Marnes-la-Coquette, France). Colistin susceptibility testing was performed with broth microdilution using SensiTest Colistin (Liofilchem, Roseto degli Abruzzi, Italy). Confirmation of ESBL phenotype was done on Mueller-Hinton agar with ceftazidim and cefotaxime discs, with and without clavulanic acid.

### 2.4. Detection of ESBL-Encoding Genes

All available ESBL-producing isolates were tested for the presence of the following ESBL-encoding genes: *bla*TEM, *bla*SHV, *bla*CTX-M group 1 (CTX-M-15), *bla*CTX-M group 2, and *bla*CTX-M group 9, by multiplex PCR following the previously described protocol [15]. The commercial Phusion U Green Multiplex PCR Master Mix (Thermo Fisher Scientific, Waltham, Massachusetts, USA) kit was utilized.

### 2.5. Infection Definition

Infection was defined as the presence of clinical signs of infection with laboratory confirmation (culture positive blood culture and/or tracheal aspirate) or post-mortem histopathological examination. Infections among colonized subjects were categorized as: (1) infection with colonizing bacteria if species identification and susceptibility patterns of rectal and blood/tracheal isolate were the same, or divergence if the susceptibility category (S-I or I-R) existed for one antimicrobial agent only; (2) infection with a microorganism other than colonizing MDR bacteria when abovementioned criteria have not been met.

### 2.6. Statistical Analysis

Statistical analysis was performed using the chi-square test or Fisher test when appropriate for comparison of categorical data. For continuous variables that were normally distributed, the *t*-test was applied, otherwise the Mann–Whitney U test was used. A two-tailed value of *p* < 0.05 was considered to be statistically significant.

### 2.7. Ethical Approval

The study was approved by the Ethical Committees of the Institute of Neonatology (No 3817/3-2017) and the Faculty of Medicine, University of Belgrade (No 1322/II/82-2020).

## 3. Results

### 3.1. Colonization with MDR Bacteria within First Week of Life and Risk Factors for Colonization

Of 103 randomly selected infants screened on admission during the first week of life, 61 were found to be colonized with MDR bacteria (11 with two different MDR bacteria); 42 were not colonized and served as a control group (Supplementary Materials Table S1). Of the 61 colonized neonates, 12 were colonized on admission, while 49 became colonized at the Institute of Neonatology. In the group of 12 neonates found to be colonized on admission, nine had spent several days at local obstetric/neonatal hospitals prior to transfer to the Institute of Neonatology and were significantly older (hours post-partum) than the other neonates (*p* < 0.01). Cesarean section (*p* = 0.04) and mechanical ventilation (*p* = 0.021) were both significant risk factors for colonization with MDR bacteria (Supplementary Materials Table S1).

### 3.2. Characteristics of MDR Bacterial Isolates

**Figure 1:**
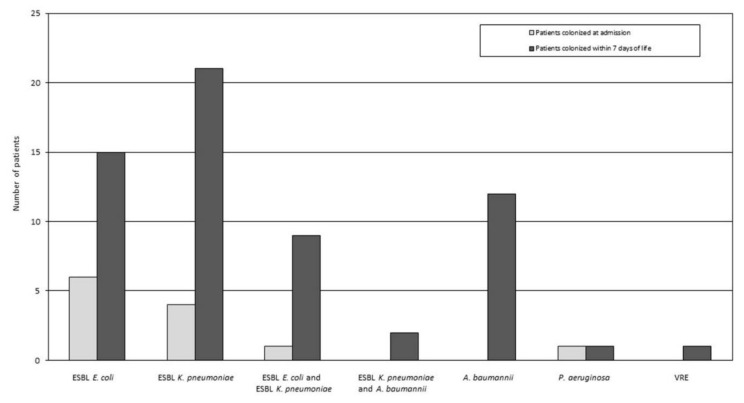
Distribution of gut colonizing multidrug-resistant (MDR) bacteria detected in preterm neonates at admission and within the first seven days of life.

A total of 72 MDR bacterial strains were isolated from 61 patients on the seventh day of life, since 11 patients harbored two different bacteria. The distribution of colonizing bacteria in neonates, detected at admission and at the seventh day of life, is presented in Figure 1. The most prevalent species was ESBL-producing *Klebsiella pneumoniae* (32/72, 44%), followed by ESBL-producing *Escherichia coli* (24/72, 33%) and *Acinetobacter baumannii* (14/72, 19%), while *Pseudomonas aeruginosa* and VRE were rare (detected in one patient each, 2%). Among patients colonized with multiple MDR bacteria, the vast majority (9 of 11) harbored ESBL-producing *E. coli* and ESBL-producing *K. pneumoniae*.

Overall, ESBL-producing enterobacteria showed uniform susceptibility to colistin (MIC 0.25–1 μg/mL) and carbapenems, while susceptibility to the remaining beta lactams and other antibiotics was variable. Resistance rates for beta lactam agents in *K. pneumoniae* isolates were the following: 73% to amoxicillin clavulanic acid, 97% to cefotaxim, 76% to ceftazidim, 65% to piperacillin-tazobactam, and 73% to cefepime. Level of resistance was high to amikacin and gentamicin (94% and 75%, respectively), and lower to fluoroquinolones (31%), trimethoprim-sulfamethoxazole (28%), and chloramphenicol (31%). In ESBL-producing *E. coli* strains, resistance rates were the following: 75% to amoxicillin clavulanic acid, 96% to cefotaxim, 83% to ceftazidim, 65% to piperacillin-tazobactam, 87% to cefepime, 100% to gentamicin, 50% to amikacin, 62% to fluoroquinolones, 50% to trimethoprim-sulfamethoxazole, and 25% to chloramphenicol. *Acinetobacter baumanni* isolates were resistant to carbapenems (100%), amikacin (100%), gentamicin (86%), fluoroquinolones (86%), and susceptible to colistin (100%) and trimethoprim-sulfamethoxazole (72%). The *Pseudomonas aeruginosa* strain found in one patient exhibited resistance to piperacillin, piperacillin-tazobactam, carbapenems and fluoroquinolones, and was susceptible to aminoglycosides, whilst a single identified VRE isolate, *E. fecium*, was resistant to ampicillin and gentamicin and susceptible to trimethoprim-sulfamethoxazole, linezolid and daptomycin. Screening for ESBL genes was available for 26/31 *K. pneumoniae* strains and 15/24 *E. coli* isolates. *bla*TEM and *bla*CTX-M-15 were predominant, followed by *bla*SHV family genes. None of the isolates harbored *bla*CTX-M-2 or *bla*CTX-M-9 group genes. *bla*TEM, *bla*CTX-M-15 and *bla*SHV were detected in 16, 15 and 12 *K. pneumoniae* isolates, respectively. Among *E. coli* isolates, *bla*TEM, *bla*SHV and *bla*CTX-M-15 were detected in 10, 8 and 7, respectively. Overall, the majority of tested isolates (25/41) carried two different ESBL genes (*bla*TEM/*bla*SHV or *bla*TEM/*bla*CTX-M-15), whereas isolates with a single resistance determinant carried either *bla*SHV or *bla*CTX-M-15 (Table 1).

### 3.3. Infections and Outcomes

Characteristics of patients who developed infections, their colonization status, and their outcomes are shown in Table 2. Overall, 9 of 103 neonates developed infection by the predefined criteria, 6 of 61 MDR-colonized patients, and 3 of 42 control patients. Eight of the nine who developed infection were successfully treated with antimicrobials, and one patient died. Of the six MDR-colonized patients who developed infections, five were due to their colonizing organisms (four *K. pneumoniae*, one *A. baumannii*), one was due to coagulase-negative staphylococci. Of the three control patients who developed infections, two were successfully treated for sepsis caused by *Streptococcus pneumoniae* or ESBL-producing *K. pneumoniae*, and one died of clinically- and histopathologically-proven sepsis of an unknown cause.

## 4. Discussion

The present study is the first one performed in Serbia focusing on gut colonization with resistant pathogens in a highly vulnerable population of preterm neonates, treated in a single tertiary healthcare center. Our results showed very high prevalence of MDR colonization of nearly 60%, with as many as 11% of patients carrying multiple MDR bacteria in the first week of life. A diversity of isolated MDR pathogens that include ESBL-producing *K. pneumoniae*, ESBL-producing *E. coli*, *A. baumannii*, *P. aeruginosa* and VRE, is particularly worrisome as these bacteria are among the major HAI-related pathogens and have propensity to cause outbreaks in hospitalized preterm neonates [16,17]. The majority of ESBL-producing enterobacterial strains were resistant to third generation and fourth generation cephalosporins, penicillins and aminoglycosides, while isolates belonging to non-fermenting Gram-negative bacilli were carbapenem resistant.

Information on the presence of the major ESBL-encoding genes in Serbia is scarce. The present study is the first exploring distribution of these genes in clinical isolates. A variety of ESBL genes were detected, with predominance of *bla*TEM and *bla*CTX-M-15, followed by *bla*SHV. The high prevalence of CTX-M-15 is expected as it is globally disseminated and prevails worldwide [18], and its presence has already been reported in Serbia among carbapenem and colistin resistant *K. pneumoniae* isolates [19]. On the other hand, the high frequency of the TEM family found in our study is surprising, since its occurrence has progressively diminished in all regions; in Europe, in a recently conducted survey, it was detected in <1% of ESBLs [20]. In addition, the overwhelming majority of tested strains of *K. pneumoniae* (16/26) and *E. coli* (10/15) carried two ESBL enzymes (*bla*TEM/*bla*CTX-M-15 or *bla*TEM/*bla*SHV) among our isolates. Such a finding is rather rare in other settings [11,21]. However, in a recent study from Tanzania, the predomination of isolates with three different ESBL enzymes in neonatal units has been demonstrated [22].

In general, prevalence of MDR colonization in hospitalized neonates varies widely, with rates in Western countries varying from 1% in USA [23], to up to 25% in Germany [9] and 30% in Italy [21]. Much higher frequencies were recorded in less developed countries such as Morocco (60%) [24], Ethiopia (74%) [25] and Madagascar (>70%) [26]. Unlike other studies, we focused on early MDR colonization in the first week of life among hospitalized preterm neonates. The observed high colonization rates in this early period implies that even higher rates would be expected upon further hospitalization if screening was done on discharge from the hospital, emphasizing the significance of our results.

Concerning the risk factors for colonization, data available so far describe the mode of delivery, younger gestational age, lower birth weight, invasive procedures, mechanical ventilation and antibiotic consumption as factors that predispose the acquisition of MDR bacteria in neonates [4], although gestational age or other parameters of maturity have not been consistently associated with colonization [11]. In our cohort, mechanical ventilation was the only parameter shown to be a significant risk factor for colonization at the study site, while neither the level of maturity (birth weight, gestational age, Apgar score etc.), nor admission to the NICU were associated with MDR bacterial carriage. Delivery by cesarean section has been shown to increase the risk of carriage of hospital-related pathogens in comparison to the vaginal delivery [27]. Our results are in line with these findings, and it might be speculated that delivery by cesarean section facilitated the rapidity of colonization with MDR bacteria, as assessed through high prevalence of colonization in the first days of life. The length of hospital stay has also been shown to be an important risk factor for MDR colonization in neonates [11,16]. In 80% (49/61) of the colonized neonates, colonization with MDR bacteria was acquired at the study site, while in 20% (12/61) it was already present on admission (12% of all patients). Most of the previously colonized neonates spent a few days at local neonatal wards and were significantly older at admission compared to other subjects, indicating prolonged prior hospitalization as the probable source of MDR colonization. Similar findings have been reported in the USA where the age of transfer to a NICU was strongly associated with colonization with MDR bacteria of interest [23]. Antibiotic exposure as a factor that affects colonization could not be reliably evaluated in this study, since initial therapy, comprising ampicillin and gentamicin/amikacin, was administered uniformly to all the patients admitted to hospital. However, a high level of resistance of the colonizing MDR bacteria to these agents implicates the probable role of antibiotic exposure in promoting and/or facilitating colonization. Indeed, in a recently performed meta-analysis exploring ESBL-producing enterobacterial colonization in a NICU, ampicillin/gentamicin therapy, mechanical ventilation and invasive procedures were identified as risk factors for colonization [28]. In addition, in a study conducted in a NICU in Spain [16], early colonization (during the first nine days of hospitalization) with ESBL-producing *K. pneumoniae* was associated with antibiotic therapy (third generation cephalosporins plus aminoglycoside).

Colonization with MDR bacteria is thought to precede infection and is considered an important risk factor for subsequent infection [6,8,10]. In the recently published meta-analysis, the frequency of infection following colonization varied between the studies from 0 to 42.8% [8]. In our cohort, the frequencies of infections in colonized patients and controls were similar (9.8% and 7.1%, respectively). However, the risk of infection due to the MDR pathogens detected in colonization appears to be higher among carriers compared to non-colonized in the present study, although the difference was not significant. Among six patients with infection due to MDR bacteria (ESBL-producing *K. pneumoniae* and *A. baumannii*), five of them had previously been colonized with these pathogens (four with *K. pneumoniae* and one with *A. baumannii*). Interestingly, though ESBL-producing *E. coli* was rather frequently present in colonization, there was no evidence of infection due to this bacterium in our study. Among the neonates that developed infection, the majority were extremely or very immature, with a body weight < 1500 g, therefore inherently being at a higher risk of infection [4,8,28], and the average time of disease onset (6–28 days) was similar to other studies [10]. Apart from colonization status, the rate of subsequent infection is probably influenced by many other factors, such as the virulence of microorganisms and adequate causal and supportive therapy. In our study, except for one patient that died from infection that was not laboratory confirmed, all other patients were effectively treated and cured. Though infections caused by ESBL-producing *K. pneumoniae* were successfully treated with meropenem, carbapenems consumption is known to facilitate the development and spread of carbapenem-resistant organisms (e.g., MDR Gram-negative non-fermenting bacilli), as might have happened among our patients.

Finally, our study has several limitations. The focus was on the colonization in the first week of life and subsequent follow-up cultures were not performed. Therefore, the likelihood of later colonization in control group patients and its relevance have not been evaluated. In addition, analysis of clonal relatedness of MDR isolates has not been assessed.

In conclusion, this study addressed the problem of MDR bacterial colonization and its association with subsequent infections in vulnerable population in a setting that lacks routine screening practices. The high colonization rate in patients transferred from other neonatal wards, as well as the high frequency of acquisition of MDR bacteria at the Institute of Neonatology, infer to inefficient preventive practices applied in neonatal wards/hospitals in Serbia. While hands and environmental hygiene and strict milk preparation practices are in place, other measures are still largely lacking, such as screening of colonized neonates in wards, and more stringent antibiotic stewardship. Therefore, alarmingly high rates of colonization, diversity and rapid dissemination of MDR bacteria found in the present study should raise awareness among health professionals and stakeholders and prompt targeted interventions to mitigate resistance selection and propagation. Lessons learned from the present example in Serbia could be useful for many other countries where similar problems exist.

## Figures and Tables

**Table 1:** Distribution of major ESBL-encoding genes among *Klebsiella pneumoniae* and *Escherichia coli* isolates.

	Isolates with One ESBL Gene (*n*, %)		Isolates with Two ESBL Genes (*n*, %)		Isolates with Three ESBL Genes (*n*, %)
	*bla*CTX-M-15	*bla*SHV	*bla*TEM and *bla*CTX-M-15	*bla*TEM and *bla*SHV	*bla*TEM, *bla*SHV and *bla*CTX-M-15
*Klebsiella pneumoniae* (*n* = 26)	5 (19.2)	5 (19.2)	9 (34.6)	6 (23.1)	1 (3.9)
*Escherichia coli* (*n* = 15)	4 (26.7)	1 (6.6)	3 (20)	7 (46.7)	0

**Table 2:** Demographic and clinical characteristics, colonization with multidrug-resistant (MDR) bacteria, causative agent, susceptibility to antimicrobial agents, antibiotic therapy and outcome in nine patients that developed infection during the neonatal period.

Patient Number	Sex (Male)	Age at Admission (h)	Gestational Week	Birth Weight (g)	1st Minute Apgar Score	Delivery by Cesarean Section	NICU Stay	MDR Colonization	Time of Disease Onset	Haemoculture	Tracheal Aspirate	Antimicrobial Susceptibility to	Therapy	Outcome
1	no	48	27	1040	3	yes	yes	yes (on 7th day)	10 days	*Klebsiella* *pneumoniae*		TZP, AK, IPM, MEM, CHL, COL	MEM	cured
2	yes	72	32	1450	6	yes	no	no	6 days	*Streptococcus* *pneumoniae*		CTX, IPM, MEM, CHL, VAN	VAN	cured
3	yes	17	25	900	4	no	yes	no	3 weeks		*Klebsiella* *pneumoniae*	IPM, MEM, CHL, COL	MEM	cured
4	yes	6	29	900	4	yes	yes	yes (on 7th day)	2 weeks	*Acinetobacter baumannii*	*Acinetobacter baumannii*	GEN, COL	MEM	cured
5	yes	14	32	1400	5	yes	yes	yes (on 7th day)	3 weeks	*Klebsiella pneumoniae*		IPM, MEM, COL	MEM	cured
6	no	18	32	1450	4	yes	yes	no	8 days	Unknown			Initial (AMP + AK)	died
7	yes	7	27	1000	3	yes	yes	yes (at admission)	3 weeks	Coagulase negative staphyloocci		FOX, VAN, CHL, FA	MEM + VAN	cured
8	no	24	31	1600	7	yes	yes	yes (at admission)	11 days	*Klebsiella* *pneumoniae*		AK, IPM, MEM, COL	MEM	cured
9	yes	50	34	1950	8	yes	no	yes (on 7th day)	6 days	*Klebsiella* *pneumoniae*		TZP, AK, IPM, MEM, COL	MEM	cured
Average ± SD		28 ± 25.54	30 ± 2.84	1299 ± 340.7	5 ± 1.66				14.3 ± 8.3					
%	66.7					88.9	77.8	66.7						11.1 (died)

Antimicrobial agents (in the order of appearance in the table): piperacillin-tazobactam (TZP), amikacin (AK), imipenem (IPM), meropenem (MEM), chloramphenicol (CHL), colistin (COL), cefotaxime (CTX), vancomycin (VAN), gentamicin (GEN), ampicillin (AMP), cefoxitin (FOX), and fusidic acid (FA); NICU—neonatal intensive care unit; SD—standard deviation.

## Data Availability

The data presented in this study are available on request from the corresponding author.

## Supplementary Materials

The following are available online at https://www.mdpi.com/article/10.3390/microorganisms9122613/s1. Table S1. Characteristics of preterm neonates colonized with multidrug-resistant (MDR) bacteria within the first week of life.
