# Supplementary material for: Colonization with Multidrug-Resistant Bacteria in the First Week of Life among Hospitalized Preterm Neonates in Serbia: Risk Factors and Outcomes
Source: Microorganisms. 2021 Dec 17;9(12):2613. doi: 10.3390/microorganisms9122613 (PMC8709168; doi:10.3390/microorganisms9122613)
Supplement: Supplementary file 1 [file microorganisms-09-02613-s001.zip › microorganisms-1456972-supplementary.pdf]

Table S1. Characteristics of preterm neonates colonized with multidrug-resistant (MDR) bacteria within the first week of life

	**Colonized at hospital admission**		**Colonized after hospital admission**		**Non-colonized**	
	N	%	N	%	N	%
Total (N=103)	12	11.6	49	47.6	42	40.8
Sex (male)	9	75	28	57.1	18	42.9
Extremely preterm (<28 weeks of gestation)	1	8.4	4	8.2	6	14.3
Very preterm (28-32 weeks of gestation)	2	16.6	19	38.8	20	47.6
Late preterm (32-37 weeks of gestation)	9	75	26	53	16	38.1
Delivery by cesarean section	10	83	34	69.4	22	52.4
Admission to the neonatal intensive care unit	3	25	25	51	23	55
Mechanical ventilation	3	25	19	38.8	10	23.8
Umbilical vein catheter	4	33.3	29	59.2	27	64.3
Birth weight (g)	1702.5 ± 343.3		1683.2 ± 497.3		1493.5 ± 498.8	
Age at admission (h)	89.8 ± 58.7		15.9 ± 19.7		19.4 ± 30.3	
1st minute Apgar score	6.8 ± 1.9		6.5 ± 1.7		5.6 ± 2.3	
